# Biomarkers of Skeletal Muscle Atrophy Based on Atrogenes Evaluation: A Systematic Review and Meta-Analysis Study

**DOI:** 10.3390/ijms26083516

**Published:** 2025-04-09

**Authors:** André Luiz Gouvêa de Souza, Anna Luisa Rosa Alves, Camila Guerra Martinez, Júlia Costa de Sousa, Eleonora Kurtenbach

**Affiliations:** 1 Instituto de Biofísica Carlos Chagas Filho, Universidade Federal do Rio de Janeiro, Rio de Janeiro 21941-902, RJ, Brazil; 2 Biosciences Applied to Health, Campus Renascença, Universidade Ceuma, São Luis 65075-120, MA, Brazil

**Keywords:** musculoskeletal, atrophy, E3 ligases, molecular research, animal models, tissue alterations

## Abstract

Muscle atrophy leads to decreased muscle mass, weakness, inactivity, and increased mortality. E3 ubiquitin ligases, key regulators of protein degradation via the ubiquitin–proteasome system, play a critical role in atrophic mechanisms. This meta-analysis followed Preferred Reporting Items for Systematic reviews and Meta-Analyses (PRISMA) guidelines, and its objective was to evaluate the association between E3 ligases Muscle Atrophy F-box (MAFbx)/Atrogin-1 (*Fbxo32*) and Muscle RING-finger protein 1 (MuRF-1) (*TRIM63*) E3 ligase mRNA levels, reductions in skeletal muscle CSA measures, and atrophy conditions. We examined papers published on PubMed^®^, Scopus, and Web of Science that studied E3 ligase gene expression signatures for *Fbxo32* (MAFbx/Atrogin-1) and *Trim63* (MuRF1) in different types of muscle atrophy and hypertrophy murine models. Twenty-nine studies selected by two independent raters were analyzed. Standardized mean differences (SMDs)/effect sizes (ESs) and 95% confidence intervals (CIs) were calculated for the outcomes using fixed-effects models. We found that 6- and 4.8-fold upregulation, respectively, of *Fbxo32* and *Trim63* was sufficient to reduce the ES to −3.89 (95% CI: −4.45 to −3.32) for the muscle fiber cross-sectional area and the development of skeletal muscle atrophy. I² and Q test statistics did not indicate heterogeneous data. There was a low probability of bias after both the funnel plot and Egger’s test analyses. These results were sustained independently of the atrophic model and muscle type. Therefore, the magnitude of the increase in muscle *Fbxo32* and *Trim63* mRNA is a feasible, reliable molecular marker for skeletal muscle atrophy in mice. The next step for the Ubiquitin-proteasome system (UPS) field involves elucidating the targets of E3 ligases, paving the way for diagnostic and treatment applications in humans.

## 1. Introduction

Skeletal muscle atrophy, characterized by a reduction in muscle mass and a decrease in its contractile capacity, is commonly identified through a reduction in the cross-sectional area (CSA) of muscle fibers [1]. Atrophy is a converging feature of several muscle–skeletal disorders, in addition to being an important determinant of the functional performance of patients with other diseases, such as cancer, sepsis, cardiomyopathies, and methylmercury poisoning [2,3]. With increasing average life expectancy, it is reasonable to predict that more individuals will suffer from muscle disorders during their lifetimes [4]. Considering the importance of skeletal muscle in ensuring autonomy and quality of life, research on morphological, functional, and metabolic changes in skeletal muscle tissue has gained prominence in the scientific community. According to the PubMed^®^ database, from 1856 to date, there are 47,627 results for articles in which the keywords “muscle atrophy” are used. However, the mechanisms that trigger muscle atrophy are under constant debate in the specialized literature [5].

Initially described as a structural change arising from an imbalance between protein synthesis and degradation, it is now known that the onset and progression of muscle atrophy is accompanied by damage to and the imbalance of energetic metabolism and redox homeostasis, factors that can culminate in the reduced viability and even death of skeletal myocytes [6]. In this context, the operation of the ubiquitin–proteasome system (UPS) has also attracted increased attention since 2001 [7].

The UPS is the largest intracellular proteolytic system, responsible for the degradation of oxidized and ubiquitinated/polyubiquitinated proteins. This refined catabolic system comprises a sequence of enzymatic reactions responsible for selecting, tagging, and degrading its target proteins. ATP and enzymes known as “E3 ligases”—the ubiquitin protein and the proteasomal multicatalytic core—are essential for this process. In simple terms, the ubiquitin protein (which functions as a marker tag) is initially activated by a thioester bond with the E1 enzyme in an ATP-dependent reaction. This activated ubiquitin is then transferred to the E2 conjugase, which promotes the conjugation of several ubiquitin molecules, forming a polyubiquitin tail. Subsequently, the E3 ligase transfers this polyubiquitin tail to the target protein that will be degraded. Finally, the target polyubiquitinated protein is recognized and degraded by the proteasome complex into small peptides [8]. Another class of enzymes called deubiquitinases can remove ubiquitin molecules from target proteins; these can prevent degradation via the proteasome and enable the recycling of these marker molecules after proteolysis [9].

In skeletal muscle, the main E3 ligases active during muscle proteolysis are MAFbx/Atrogin-1 and MuRF-1, which tag myofibrillar proteins to be degraded [6]. Many studies have investigated possible changes related to the activity and/or gene expression of the UPS’s components in several pathological conditions that lead to muscle atrophy. MuRF1 preferentially targets proteins of the Z line structuring complex, such as vimentin, desmin, and α-actinin [10]. This E3 ligase also catalyzes the ubiquitination of proteins present in the thick filament, the light and heavy chains of myosin, and MyBPC [11]. MAFbx appears to target pro-anabolic factors like MyoD, myogenin, and eIF3f [10]. Decreases in expression and increases in the degradation of these proteins provoke sarcomere myofibrillar disruption, resulting in severe contractile dysfunctions.

In this study, we present the results of a comprehensive meta-analysis comparing the gene expression levels of MAFbx/Atrogin-1 and MuRF-1 between atrophic and non-atrophic mice. The objective was to evaluate the association between MAFbx/Atrogin-1 (*Fbxo32*) and MuRF-1 (*TRIM63*) E3 ligase mRNA levels, reductions in skeletal muscle CSA measures, and atrophy conditions. Our study was meticulously designed to establish a sensitive, reproducible, and quantitative molecular marker for skeletal muscle atrophy.

## 2. Materials and Methods

This meta-analysis was developed in accordance with PRISMA [12] (see the checklist in Table 1) and registered with the INPLASY database (INPLASY202520089).

### 2.1. Criteria for Eligibility

#### 2.1.1. Types of Studies and Interventions

Randomized, non-randomized, and controlled studies published in the English language and available on the PubMed^®^, Scopus, and Web of Science databases were selected for inclusion.

#### 2.1.2. Characteristics of the Animal Model

The animals included in the studies were male mice, aged between 4 and 100 weeks, subjected to muscle atrophy induction protocols. In addition, only male mice were included to minimize sex-related variability and ensure data homogeneity across studies.

#### 2.1.3. Characteristics of the Variables Measured

The current meta-analysis only included studies that analyzed mouse skeletal muscle CSA via histology. The staining methods were as follows: hematoxylin and eosin (H&E); laminin-stained immunohistochemistry; and dye-ATPase. The *Fbxo32* (Atrogin/MAFbx gene) and *Trim63* (MuRF1 gene) mRNA expression levels were determined using polymerase chain reaction in real time (qPCR) or Northern blotting.

### 2.2. Selection Strategy

The studies were selected without a publication date limitation. The following keywords were used in combination: “E3 ligases and muscle atrophy” and “E3 ligases and muscle hypertrophy”.

### 2.3. Study Selection

The search generated 584 studies that were reviewed by three researchers who were responsible for selecting the studies for subsequent analysis. A round of title and abstract screening was followed by full-text screening. All three reviewers independently assessed all articles and then reached a consensus on their inclusion. A fourth experienced researcher was responsible for settling any disagreements between the three reviewers, when necessary.

**Figure 1:**
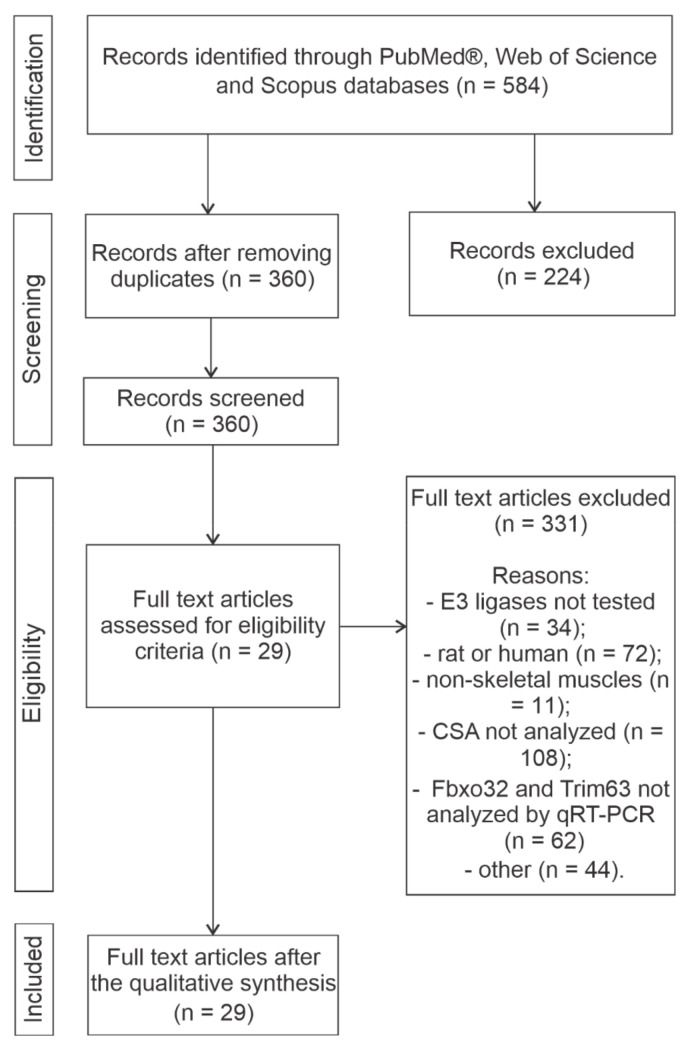
Flow diagram of the systematization process for inclusion of articles in this meta-analysis. The data for all articles included were quantitatively acquired and analyzed.

Figure 1 presents the identification process for selecting the number of studies included in the analysis flow chart. Using this selection strategy, 584 studies were retrieved. After removing duplicates, 360 were screened. In total, 330 studies were excluded after applying the inclusion criteria, such as E3 ligases untested, studies not performed on mouse skeletal muscle, and CSA not analyzed. Another 44 studies were excluded for reasons such as retraction due to failure, female mice, or CSA and qPCR obtained from different muscles. Finally, 29 [13,14,15,16,17,18,19,20,21,22,23,24,25,26,27,28,29,30,31,32,33,34,35,36,37,38,39,40,41] out of the 584 studies were selected for analysis based on the eligibility and inclusion criteria.

### 2.4. Data Extraction

The mean and standard deviation values of CSA, *Fbxo32* (Atrogin/MAFbx gene) mRNA levels, and *Trim63* (MuRF1 gene) mRNA levels in the control and treatment groups were extracted from the studies using a dimensional tool for graphical analysis (CorelDRAW^®^, Graphics Suite, version 12.0 for Windows). When it was impossible to obtain data from the graphs for a certain result, the result was not included in the analysis.

### 2.5. Bias Risk Assessment and Degree of Between-Rater Agreements

A funnel plot was used to visualize the data symmetry, and Egger’s test was used to determine the bias risk [42]. Two researchers analyzed the quality level of the studies utilizing the NIH-Study Quality Assessment Tool for controlled intervention studies [43].

### 2.6. Consistency of the Selected Studies

The heterogeneity of the selected studies was analyzed using the Cochran Q test [44] and I2 statistics [45]:

I2 = (Q − df)/Q × 100%(1)

where Q is the Cochran Q test, and df is the degrees of freedom.

### 2.7. Effect Size

The pooled effect size (ES) (also represented as the standardized mean difference (SMD) and standard deviation (SD)) was calculated from the average, SD, and sample size (n) of the control and treatment groups. A pooled analysis of the estimated ranges was developed with a fixed-effects model performed using STATA 10.0 (StataCorp LP, College Station, TX, USA). In addition, the fixed-effect model was chosen due to the small number of studies, ensuring a stable estimation.

#### Subgroup Analysis

After establishing the ES of the studies, which was ordered ascendingly, a quartile division was employed considering the magnitude of the ES of CSA change across each study. A set of subgroup studies were scrutinized for each quartile. For each quartile, we calculated the *Fbxo32* and *Trim63* mRNA differences between the control and treatment groups, expressed in multiples.

## 3. Results

### 3.1. Main Characteristics and Quality of the Selected Studies

The quality assessment of the 29 publications was conducted using the NIH-scale tool for controlled intervention studies. Twenty-one studies were rated as good quality, while eight were rated fair, and all were included.

Table 2 shows the *Fbxo32* and *Trim63* mRNA expression levels and the measurements of the CSA of skeletal muscle tissue from the 289 male mice described in the 29 studies, corresponding to different experimental conditions, totaling 48 cohorts. Several skeletal muscle types were considered, as well as different experimental conditions that lead to muscle atrophy or hypertrophy. Twenty-six studies showed a decrease and three showed increases in the CSA of the mouse skeletal muscle cells relative to the controls after applying the experimental protocols (the general CSA average was ↓ 26.8% ± 21.1). The *Fbxo32* and *Trim63* mRNA expression levels in muscle homogenates were analyzed using qRT-PCR in all studies. The results showed a 2.6 ± 5.1 times increase in the average for *Fbxo32* and 2.4 ± 4.7 times for *Trim63* relative to the controls. Notably, greater E3 ligase increases were observed in cancer cachexia [29], lung injuries [36], and burn [28] injuries with muscle atrophy protocols.

### 3.2. Main Effect, Heterogeneity and Risk of Bias in the Studies

**Figure A1:**
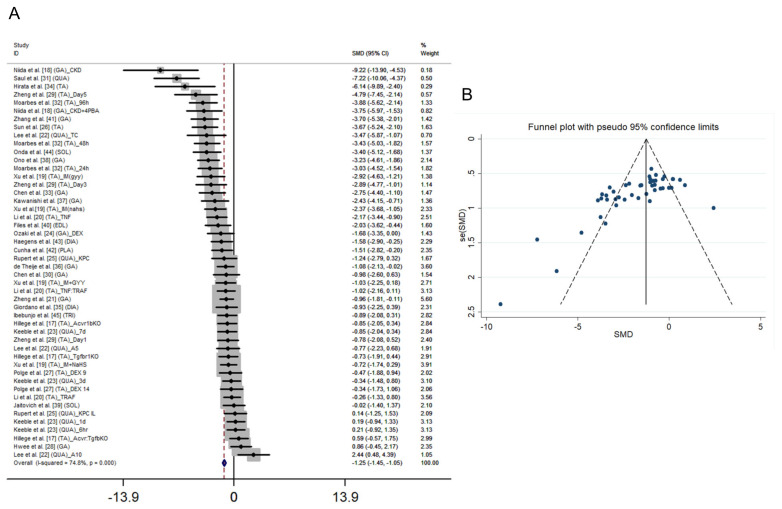
Main Effect, Heterogeneity and Risk of Bias in the Studies. (**A**) General forest plots of mouse skeletal muscle cross-sectional area data from 29 studies and 48 cohorts. The effect sizes (ESs = SMD (standardized mean difference)) and 95% confidence intervals (CIs) of the studies are stratified according to the percentage variations in CSA observed between experimental and control groups. Overall ESs are represented by the diamond on the horizontal x-axis. (**B**) Funnel plots of ES (standardized mean difference (SMD)) against standard error deviation (SE (SMD)). A pseudo 95% confidence interval is represented by dotted lines [13,14,15,16,17,18,19,20,21,22,23,24,25,26,27,28,29,30,31,32,33,34,35,36,37,38,39,40,41].

The average SD of the CSA and the sample size of the control and treatment groups of all 48 cohorts were considered for analysis (the raw data are detailed in Appendix B). The ES shows the magnitude of changes in the skeletal muscle CSA, with negative effects indicating muscle atrophy, while positive effects show a muscle hypertrophy tendency. Mice belonging to the treatment groups reduced CSA at the expense of muscle atrophy (ES = −1.25; 95% CI: −1.45 to −1.05) (Appendix A: Figure A1A). Cochran’s Q test (x^2^) and inconsistency I^2^ statistics confirmed the heterogeneity of the data (x^2^ = 0.000 and I^2^ = 74.8%). The risk of bias was analyzed via funnel plot, and visual asymmetry was noted. The publication bias was confirmed with Egger’s test (*p* = 0.0001) (Appendix A: Figure A1B).

**Figure 2:**
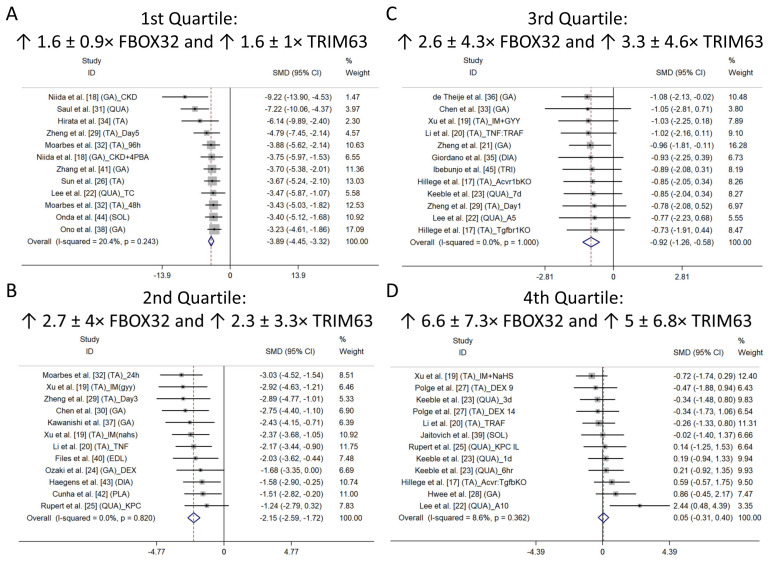
Forest plots of mouse skeletal muscle cross-section area data from 29 studies and 49 cohorts. The effect sizes (ES = SMD (standardized mean difference)) and 95% confidence intervals (CIs) of the studies are stratified into quartiles according to the percentage variations in CSA observed between the experimental and control groups. (**A**) First, (**B**) second, (**C**) third, and (**D**) fourth quartiles. Overall ESs are represented by the diamond on the horizontal x-axis. The headings of the graphs indicate the average change in *Fbxo32* and *Trim63* RNAm muscle levels, comparing experimental to control groups [13,14,15,16,17,18,19,20,21,22,23,24,25,26,27,28,29,30,31,32,33,34,35,36,37,38,39,40,41].

To better comprehend the nature of heterogeneity when the data were analyzed together and to demonstrate the extent of the influence of *Fbxo32* and *Trim63* expression on muscle CSA atrophy, a subgroup analysis was performed. Therefore, a quartile division was employed considering the ES magnitude of CSA change across each study. A set of subgroup studies was scrutinized for each quartile (quartile 1: ES −9.21 to −3.23; quartile 2: ES −3.02 to −1.23; quartile 3: −1.07 to −0.73; and quartile 4: −0.72 to +2.44) (Figure 2). In addition, for each quartile, the average variation values (in times) of *Fbxo32* and *Trim63* mRNA were determined by comparing experimental to control groups. In general, when there was a substantial decrease in CSA muscle, increases of at least 6 ± 6.5× in *Fbxo32* and 4.8 ± 4.1× *Trim63* mRNA levels were observed compared with the values of the control condition. The pooled ES of the change in muscle CSA (n = 81 mice) was −3.89 (95% CI: −4.45 to −3.32) (Figure 2A). There was no evidence of data heterogeneity (I2 = 20.4%; *p* = 0.243) in the 10 studies (12 cohorts) used for this analysis. In the second quartile, the *Fbxo32* and *Trim63* levels increased by about 7.7 ± 5.9 and 7.9 ± 6.9 times, respectively, with a decreasing CSA ES (n = 71) of −2.15 (95% CI: −2.59 to −1.72) (Figure 2B). The data did not demonstrate heterogeneity in 12 cohorts from the 11 studies in this quartile (I2 = 0%; *p* = 0.82). In quartile three, *Fbxo32* varied by 1.6 ± 1.9 times and *Trim63* 2.1 ± 1.2 times and showed an ES of −0.92 (CI: −1.26 to −0.58) (n = 73 mice) (Figure 2C). The data for this quartile did not demonstrate heterogeneity for 12 cohorts from the 10 studies analyzed (I2 = 0%; *p* = 1.00). In the last quartile, *Fbxo32* and *Trim63* RNAm levels increased by 1.8 ± 1.4 and 1.8 ± 1.4 times, respectively, with no change in muscle CSA, as shown by an ES of 0.05 (95% CI: −0.31 to 0.4) (n = 63 mice) (Figure 2D). The data from the 12 cohorts (from the 10 studies) did not demonstrate heterogeneity, which was confirmed via statistical analysis (I2 = 8.6%; *p* = 0.362).

**Figure 3:**
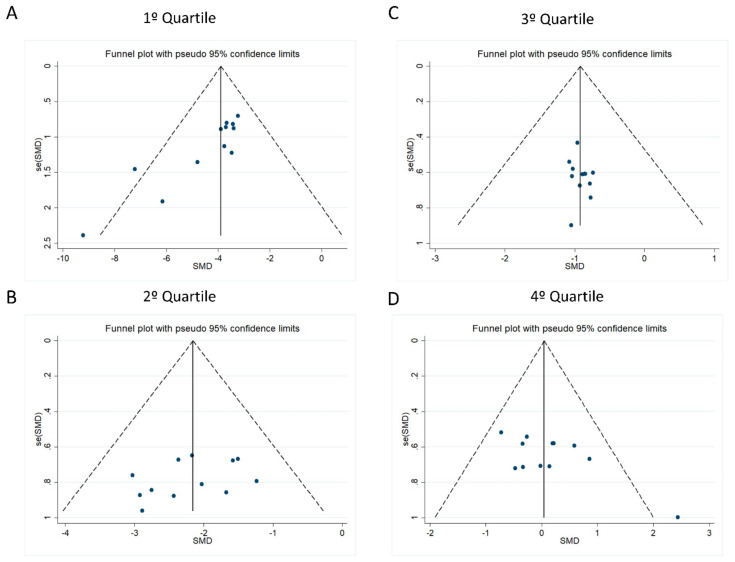
Funnel plots of ES (standardized mean difference (SMD)) against standard error deviation (SE (SMD)) for the (**A**) first, (**B**) second, (**C**) third, and (**D**) fourth quartiles. A pseudo-95% confidence interval is represented by dotted lines.

To understand the inclusion trends in studies with specific results (i.e., the risk of publication bias), they were analyzed via funnel plot graphs and Egger’s test. Visually, the data did not show asymmetry for any quartiles (Figure 3). However, Egger’s test confirmed the absence of risk of bias in the second and third quartiles (2nd: r2 = 0.17 and *p* = 0.18; 3rd: r2 = 0.02 and *p* = 0.68) and its presence in the first and fourth (1st: r2 = 0.68 and *p* < 0.001; 4th: r2 = 0.43 and *p* = 0.02).

With the systematization and meticulous choices of the inclusion criteria, these results established that increases of approximately 6 and 4.8 times in muscle *Fbxo32* and *Trim63* mRNA levels, respectively, are reliable molecular predictors for skeletal muscle atrophy in the mice models studied compared with non-atrophy conditions.

## 4. Discussion

We employed a meta-analysis to examine the mRNA levels of *Fbxo32* and *Trim63* in the skeletal muscle of mice subjected to various atrophic and hypertrophic protocols. Our findings substantiate the hypothesis that the upregulation of mRNA for both E3 ligases is associated with a reduction in the cross-sectional area of muscle tissue cells in mice.

Overall, atrophy is characterized by a loss of contractile proteins [46] associated with a reduction in muscle mass or a decrease in cellular content of approximately 5–10% [1]. Predominantly, the force generated by a muscle is a combination of muscle size and fiber type. These parameters can be assessed using well-established protocols in the literature [47]. Histology—specifically, the CSA—is a valid, direct, and reproducible measure and is the gold-standard technique for assessing muscle cell size [48,49]. Additionally, the CSA is one of the primary predictors of force production in both human and rodent models [50,51]. Therefore, the CSA emerges as a reliable parameter for distinguishing between normal and pathological states in skeletal muscle.

In this meta-analysis, we evaluated the results of articles published in the PubMed^®^, Scopus, and Web of Science databases. They were accessed using the keywords established in the Section 2, with no specified time limit for publication. Initially, 584 studies were identified, and after applying the inclusion/exclusion criteria, 29 were included in this analysis.

In a quality assessment of the 29 publications, 21 were rated good, while 8 were rated fair, and all were included. Most of the eight studies classified as “fair” were downgraded in quality based on the “similar background treatments” criterion of the NIH scale for controlled intervention studies. This was because the animals in the control groups did not receive inactive treatments that simulated active conditions, such as a placebo or sham treatment.

The 26 studies included in our analysis demonstrated significant muscle atrophy in the modeled mice, evidenced by a reduction in CSA ranging from 5.1% to 77.3%, with a mean of 32.9%. Including the term “hypertrophy” in the search strategy was crucial to minimizing the risk of publication bias. This approach ensured a more comprehensive dataset and reduced the likelihood of selectively capturing only studies that directly associated E3 ligases with muscle atrophy, thereby enhancing the reliability and balance of the meta-analysis findings. Five studies revealed a significant increase in CSA, indicating hypertrophy. However, only one cohort from the study by Lee et al. [18] demonstrated a substantial effect size (ES), as illustrated in Figure 2D. Conversely, none of the cohorts associated with hypertrophy exhibited a significant increase in E3 ligase mRNA. Notably, other analyses were rendered impractical due to the limited number of studies on additional E3 ligases that have also been associated with muscle atrophy, such as MuRF-2/3, Nedd4, and MUSA1 [52,53].

In an initial analysis involving all 29 studies, we observed an average increase equal to twice that of the *Fbxo32* and *Trim63* mRNA levels associated with muscle CSA reduction, with an effect size of −1.25 (CI: −1.45 to −1.05). However, these results were accompanied by significant heterogeneity (Q test, *p* = 0.000), substantial inconsistency among studies (I2 = 74.8%), and evidence of publication bias (Egger’s test, *p* = 0.0001). It is crucial to acknowledge the nature of this systematic review and meta-analysis study, recognizing that despite stringent systematic criteria, there may still be methodological limitations, including variations among the included studies. A subgroup analysis was conducted by dividing the studies into quartiles, aiming to enhance our understanding of the observed heterogeneity in the overall data analysis. The impact of *Fbxo32* and *Trim63* expression levels on muscle CSA atrophy was further examined in this analysis. With the division into subgroups by quartile, the data heterogeneity was resolved. Elevated levels of E3 ligase genes were associated with lower CSA levels (refer to Figure 2A–D). Consistent with previous findings [32,41], our results showed that the onset of muscle atrophy did not occur when the increase in the *Fbxo32* and *Trim63* E3 ligases’ mRNA content was greater than twice that of the housekeeping gene.

While none of the quartiles exhibited visual asymmetry in the funnel plot, Egger’s test detected significant bias (*p* < 0.05) in the first and fourth quartiles. In the third quartile, an absence effect was visualized due to the confidence interval of most studies crossing the zero vertical axis. Notably, all quartiles displayed points outside the confidence interval limits (refer to Figure 3A,D), and the publication bias (via Egger’s test) was resolved after excluding these studies. This bias may be attributed to the tendency of most published studies to report only significant results, variations in study quality, and small sample sizes [54].

In addition, we conducted an additional subgroup analysis considering studies that had investigated the same skeletal muscles (gastrocnemius, quadriceps, and tibialis anterior) and another analysis with the same atrophy protocols (cancer cachexia and dexamethasone protocols). All of these subgroups showed a negative effect on CSA size; however, significant heterogeneity, substantial inconsistency, and bias were observed. We conducted an additional analysis of the effect size of CSA based on variations in *Fbxo32* and *Trim63* levels. Although an increase in both E3 ligases was associated with a decrease in CSA size, significant heterogeneity, considerable inconsistency, and potential bias were observed.

MuRF-1 directly targets contractile proteins for degradation (α-actin, MHC, etc.), while MAFbx appears to target pro-anabolic factors such as MyoD, myogenin, and eIF3f [55]. Both are regulated by the same transcriptional factors (FOXO1/FOXO3a, NF-κB, C/EBP β, Smad 3, etc.) and signaling pathways [56,57]. Although their specific in vivo substrates are yet to be fully elucidated, MuRF1 and Atrogin-1/MAFbx knockout mice show resistance to muscle atrophy induced by denervation [6], suggesting that these two genes play crucial roles as regulators of muscle atrophy. The regulation of the UPS, particularly in controlling the level of E3 ligases, could represent a promising strategy for preventing or even ameliorating muscle atrophy. Protein degradation induced by the ubiquitin–proteasome system (UPS) is intricately linked with the pathophysiological mechanisms of muscle atrophy. Consequently, protease inhibitors emerge as a potential therapeutic strategy [58,59]. The administration of MG-132 and Velcade, two proteasome inhibitors, restored normal protein levels in mdx mice, a model of Duchenne muscular dystrophy (DMD). Notably, while MG-132 inhibits both proteasome and calpain activity, Velcade exhibits a selective and high affinity for the proteasome. However, when freshly isolated skeletal muscle biopsies from DMD patients were treated with these drugs, signs of phenotype rescue were not universally observed in all examined explants [58,60]. Furthermore, the promising histological effects resulting from proteasome activity inhibition do not necessarily translate into functional benefits and may induce muscle dysfunction and weakness [61]. Although inhibiting proteolysis to prevent atrophy appears to be a promising strategy, the associated risks need to be carefully considered.

To the best of our knowledge, there is a limited body of research dedicated to E3 ligase inhibition for atrophy treatment, with specific inhibitors of MuRF-1 having been explored in only a few studies [62]. None have been reported for MAFbx. However, advancements in in vitro and in silico screening methods have facilitated the discovery of several compounds targeting components of the UPS, some of which have progressed to clinical trials [63,64]. Recently, novel small molecules acting as inhibitors of MuRF1 and Muscle RING-finger protein-2 (MuRF2) were administered to rodents, demonstrating the reversal of muscle wasting across different atrophy protocols [65]. This innovative strategy is based on inhibiting the interaction between titin and MuRF1, thereby protecting myofibrils from extensive proteolysis via the UPS. Future studies will likely be conducted using diverse atrophy protocols and extended follow-up periods to widely assess the efficacy and safety of these compounds. These findings offer more precise tools for the scientific community to associate muscle atrophy genes compared with those available since their characterization in 2001 [6]. In recent years, significant attention has been devoted to elucidating the molecular mechanisms underlying atrophy, with the UPS emerging as a key player in mediating muscle wasting. Our results demonstrate that increases in the mRNA levels of *Fbxo32* and *Trim63* E3 ligases are sufficient to manifest a reduction in the CSA, the gold standard for detecting muscle atrophy.

Until now, therapeutic trials have primarily relied on proteasome inhibitors. Only a few research groups are actively developing drugs capable of inhibiting or depleting E3 ligases. The intricate nature of the ubiquitination reaction and the challenge of selectively inhibiting one E3–ubiquitin–protein interaction over others present considerable hurdles. However, achieving this could yield maximum therapeutic benefits with minimal toxicity. Therefore, the magnitude of the increase in muscle *Fbxo32* and *Trim63* mRNA is a feasible, reliable molecular marker for skeletal muscle atrophy in mice. The next step for the UPS field involves elucidating the targets of E3 ligases, paving the way for diagnostic and treatment applications.

## Figures and Tables

**Table 1:** PRISMA checklist.

Title			
Title	1	Identify the report as a systematic review.	Lines 2 to 3
**Abstract**			
Abstract	2	See the PRISMA 2020 for Abstracts checklist.	Lines 10 to 25
**Introduction**			
Rationale	3	Describe the rationale for the review in the context of existing knowledge.	Lines 49 to 73
Objectives	4	Provide an explicit statement of the objective(s) or question(s) the review addresses.	Lines 74 to 79
**Methods**			
Eligibility criteria	5	Specify the inclusion and exclusion criteria for the review and how studies were grouped for the syntheses.	Lines 85 to 98
Information sources	6	Specify all databases, registers, websites, organisations, reference lists and other sources searched or consulted to identify studies. Specify the date when each source was last searched or consulted.	Lines 87 to 89
Search strategy	7	Present the full search strategies for all databases, registers and websites, including any filters and limits used.	Lines 100 to 102
Selection process	8	Specify the methods used to decide whether a study met the inclusion criteria of the review, including how many reviewers screened each record and each report retrieved, whether they worked independently, and if applicable, details of automation tools used in the process.	Lines 103 to 115
Data collection process	9	Specify the methods used to collect data from reports, including how many reviewers collected data from each report, whether they worked independently, any processes for obtaining or confirming data from study investigators, and if applicable, details of automation tools used in the process.	Lines 121 to 125
Data items	10a	List and define all outcomes for which data were sought. Specify whether all results that were compatible with each outcome domain in each study were sought (e.g., for all measures, time points, analyses), and if not, the methods used to decide which results to collect.	Lines 110 to 114
	10b	List and define all other variables for which data were sought (e.g., participant and intervention characteristics, funding sources). Describe any assumptions made about any missing or unclear information.	Lines 90 to 98
Study risk of bias assessment	11	Specify the methods used to assess risk of bias in the included studies, including details of the tool(s) used, how many reviewers assessed each study, and whether they worked independently, and if applicable, details of automation tools used in the process.	Lines 126 to 129
Effect measures	12	Specify for each outcome the effect measure(s) (e.g., risk ratio, mean difference) used in the synthesis or presentation of results.	Lines 138 to 142
Synthesis methods	13a	Describe the processes used to decide which studies were eligible for each synthesis (e.g., tabulating the study intervention characteristics and comparing against the planned groups for each synthesis (item #5)).	Lines 85 to 120
	13b	Describe any methods required to prepare the data for presentation or synthesis, such as the handling of missing summary statistics, or data conversions.	Lines 122 to 125
	13c	Describe any methods used to tabulate or visually display results of individual studies and syntheses.	Lines 122 to 125
	13d	Describe any methods used to synthesize results and provide a rationale for the choice(s). If meta-analysis was performed, describe the model(s), method(s) to identify the presence and extent of statistical heterogeneity, and software package(s) used.	Lines 138 to 142
	13e	Describe any methods used to explore possible causes of heterogeneity among study results (e.g., subgroup analysis, meta-regression).	Lines 131 to 136
	13f	Describe any sensitivity analyses conducted to assess robustness of the synthesized results.	Lines 143 to 146
Reporting bias assessment	14	Describe any methods used to assess risk of bias due to missing results in a synthesis (arising from reporting biases).	Lines 126 to 129
Certainty assessment	15	Describe any methods used to assess certainty (or confidence) in the body of evidence for an outcome.	Lines 137 to 142
**Results**			
Study selection	16a	Describe the results of the search and selection process, from the number of records identified in the search to the number of studies included in the review, ideally using a flow diagram.	Lines 108 to 120 and Section 2.3
	16b	Cite studies that might appear to meet the inclusion criteria, but which were excluded, and explain why they were excluded.	Lines 110 to 114
Study characteristics	17	Cite each included study and present its characteristics.	Lines 152 to 165 and Section 3.1
Risk of bias in studies	18	Present assessments of risk of bias for each included study.	Lines 208 to 218 and Section 3.2
Results of individual studies	19	For all outcomes, present, for each study (a) summary statistics for each group (where appropriate) and (b) an effect estimate and its precision (e.g., confidence/credible interval), ideally using structured tables or plots.	Lines 176 to 207 and Section 3.2
Results of syntheses	20a	For each synthesis, briefly summarise the characteristics and risk of bias among contributing studies.	Lines 167 to 175 and Figure A1B
	20b	Present results of all statistical syntheses conducted. If meta-analysis was performed, present for each the summary estimate and its precision (e.g., confidence/credible interval) and measures of statistical heterogeneity. If comparing groups, describe the direction of the effect.	Lines 113 to 124 and Figure A1A
	20c	Present results of all investigations of possible causes of heterogeneity among study results.	Lines 167 to 173 and Figure A1A
	20d	Present results of all sensitivity analyses conducted to assess the robustness of the synthesized results.	Lines 167 to 173 Figure A1A
Reporting biases	21	Present assessments of risk of bias due to missing results (arising from reporting biases) for each synthesis assessed.	Lines 173 to 175 Figure A1B
Certainty of evidence	22	Present assessments of certainty (or confidence) in the body of evidence for each outcome assessed.	Lines 176 to 207 Section 2.3
**Discussion**			
Discussion	23a	Provide a general interpretation of the results in the context of other evidence.	Lines 230 to 243
	23b	Discuss any limitations of the evidence included in the review.	Lines 192 to 194
	23c	Discuss any limitations of the review processes used.	Lines 258 to 263 and 281 to 283.
	23d	Discuss implications of the results for practice, policy, and future research.	Lines 272 to 278
**Other Information**			
Registration and protocol	24a	Provide registration information for the review, including the registered name and registration number (or state that the review was not registered).	not registered
	24b	Indicate where the review protocol can be accessed, or state that a protocol was not prepared.	not registered
	24c	Describe and explain any amendments to information provided at registration or in the protocol.	not registered
Support	25	Describe sources of financial or non-financial support for the review, and the role of the funders or sponsors in the review.	Lines 340 to 342
Competing interests	26	Declare any competing interests of review authors.	Line 351 to 352
Availability of data, code and other materials	27	Report which of the following are publicly available and where they can be found: template data collection forms; data extracted from included studies; data used for all analyses; analytic code; any other materials used in the review.	Upon request of the reviewer

**Table 2:** Characteristics of studies selected from the literature search and included for analysis.

No.	Study	n	Age (wks)	Muscle Type	Muscle Atrophy Protocol	Time Course (Days)	E3 Ligases Modulation (Times)		CSA (%)
							MAFbx (*Fbxo32*)	MuRF1 (*Trim63*)	
1	Polge et al. [23] (DEX 9)	4	20	TA	Dexamethasone treatment	9	↑ 2.7×	↑ 2.4×	↓ 26.8
	Polge et al. [23] (DEX 14)	4				14	↑ 2.6×	↑ 1.8×	↓ 22.4
2	Chen et al. [26]	6	4	GAS	Cancer cachexia	21	↑ 13.6×	↑ 9.7×	↓ 54.4
3	Chen et al. [29]	3	6–8	GAS	Cancer cachexia	15	↑ 3.9×	↑ 3.6×	↓ 58.5
4	Cunha et al. [38]	6	28	PLA	Heart failure	210	↑ 1.6×	↑ 1.3×	↓ 29.8
5	de Theije et al. [32]	8	20	GAS	Hypoxia	21	↑ 3.3×	↑ 2.3×	↓ 14.9
6	Files et al. [36]	5	8	EDL	Lung injury	3	↑ 16.3×	↑ 12.5×	↓ 23.8
7	Giordano et al. [31]	5	8	DIA	Hypoxia	4	↑ 1.2×	↑ 1.1×	↓ 28.9
8	Haegens et al. [39]	6	20	DIA	Lung inflammation	1	↑ 2.6×	↑ 5.4×	↓ 17.4
9	Hillege et al. [13] (Tgfbr1KO)	6	6	TA	Cardiotoxin-induced injury	4	↑ 1×	↑ 1×	↓ 30.6
	Hillege et al. [13] (Acvr1bKO)	6					↑ 1×	↑ 1×	↓ 22.0
	Hillege et al. [13] (Acvr:TgfbKO)	6					↑ 3.3×	↑ 1.5×	↑ 25.7
10	Hirata et al. [30]	4	10	TA	Diabetes mellitus	21	↑ 1.3×	↑ 1.3×	↓ 38.8
11	Hwee et al. [24]	5	26 to 100	GAS	Denervation	7–14	↑ 1.8×	↑ 0.04×	↑ 39.6
12	Ibebunjo et al. [41]	6	16	TRI	Orchidectomy	28	↑ 1×	↑ 2.3×	↓ 13.2
13	Jaitovich et al. [35]	4	14–16	SOL	Denervation and dexamethasone treatment	21	-	↑ 4.6×	↓ 41.2
14	Kawanishi et al. [33]	6	10	GAS	Immobilization	14	↑ 6.3×	↑ 4.5×	↓ 40
15	Keeble et al. [19] (6 h)	6	14–21	QUA	Anterior cruciate ligament tear injury	0.25	↑ 0.9×	↑ 0.9×	↑ 4.5
	Keeble et al. [19] (1 d)	6				1	↑ 1.6×	↑ 1.6×	↑ 5.9
	Keeble et al. [19] (3 d)	6				3	↑ 2×	↑ 3.3×	↓ 10.3
	Keeble et al. [19] (7 d)	6				7	↑ 0.9×	↑ 1.2×	↓ 8.8
16	Lee et al. [18] (TC)	4	6	QUA	Cancer cachexia	15	↑ 5×	↑ 7.5×	↓ 18
	Lee et al. [18] (A5)	4					↑ 1.8×	↑ 2.5×	↓ 5.1
	Lee et al. [18] (A10)	4					↑ 1.3×	↑ 1.7×	↑ 18.8
17	Li et al. [16] (TNF)	8	10 to 77	TA	Aging	5	↑ 2.1×	↑ 4.2×	↓ 60.6
	Li et al. [16] (TRAF)	8					↑ 0.9×	↑ 2.7×	↓ 8.7
	Li et al. [16] (TNF:TRAF)	8					↑ 0.7×	↑ 1.6×	↓ 30.3
18	Moarbes et al. [28] (24 h)	8	8	TA	Burn injury	1	↑ 15.8×	↑ 24×	↓ 44.8
	Moarbes et al. [28] (48 h)	8				2	↑ 7.3×	↑ 8.3×	↓ 37.4
	Moarbes et al. [28] (96 h)	8				4	↑ 25×	↑ 14.5×	↓ 31.3
19	Niida et al. [14] (CKD)	5	8	GAS	Chronic kidney disease	42	↑ 2.6×	↑ 3.3×	↓ 43.7
	Niida et al. [14] (CKD + 4PBA)	5					↑ 1.4×	↑ 1.7×	↓ 23.4
20	Onda et al. [40]	7	8	SOL	Immobilization	14	↑ 3×	↑ 2.4×	↓ 44
21	Ono et al. [34]	10	8 to 10	GAS	Diabetes mellitus	14	↑ 5.3×	↑ 3.3×	↓ 40.4
22	Ozaki et al. [20]	4	8 to 10	GAS	Dexamethasone treatment	14	↑ 2.9×	↑ 2.8×	↓ 22.6
23	Rupert et al. [21] (KPC)	4	8	QUA	Cancer cachexia	17	↑ 15.6×	↑ 17.8×	↓ 32.2
	Rupert et al. [21] (KPC IL)	4					↑ 3.2×	↑ 1.3×	↑ 4.8
24	Saul et al. [27]	8	10	QUA	Colitis	14	↑ 6.8×	↑ 5.2×	↓ 25.9
25	Sun et al. [22]	9	29	TA	Dexamethasone treatment	14	↑ 3.1×	↑ 3.8×	↓ 27.1
26	Xu et al. [15] (IM(gyy))	6	10	TA	Immobilization	14	↑ 6.3×	↑ 4.6×	↓ 44
	Xu et al. [15] (IM(nahs))	6					↑ 5.8×	↑ 4.2×	↓ 43.6
	Xu et al. [15] (IM + GYY)	6					↑ 2.9×	↑ 2×	↓ 16.9
	Xu et al. [15] (IM + NaHS)	6					↑ 2.2×	↑ 2.2×	↓ 17.8
27	Zhang et al. [37]	8	6 to 8	GAS	Cancer cachexia	19	↑ 9.3×	↑ 7×	↓ 49.1
28	Zheng et al. [17]	12		GAS	Angiotensin infusion		↑ 1.5×	↑ 1.6×	↓ 9.1
29	Zheng et al. [25] (Day 1)	5	8–10	TA	Sepsis	1	↑ 4.6×	↑ 4.7×	↓ 10.5
	Zheng et al. [25] (Day 3)	5				3	↑ 3.8×	↑ 3.4×	↓ 44.8
	Zheng et al. [25] (Day 5)	5				7	↑ 1.5×	↑ 0.9×	↓ 46.2

CSA—Cross-sectiona area; GAS—gastrocnemius; PLA—plantaris; EDL—extensor digitorum longus; DIA—diaphragm; TA—tibialis anterior; TRI—triceps; SOL—soleus; QUA—quadriceps; Tgfbr1KO/Acvr1bKO/Acvr:TgfbKO—mouse lines that Tgfbr1, Acvr1b or both receptors were deleted; h—hours; d—days; CKD/CKD + 4PBA—chronic kidney disease mice group that were given a modified diet with or without 4-PBA; KPC/KPC IL—mice who underwent orthotopic implantation of KPC or KPC IL6KO cells; IM + GYY/IM + NaHS—mice who were immobilized and injected with GYY4137 or NaHS; IM(gyy)/IM(nahs)—mice who were immobilized and utilized as the control group for IM + GYY or IM + NaHS.

## Abbreviations

The following abbreviations are used in this manuscript:

CSA—Cross-section Area; GAS—Gastrocnemius; PLA—Plantaris; EDL—Extensor Digitorum Longus; DIA—Diaphragm; TA—Tibialis Anterior; TRI—Triceps; SOL—Soleus; QUA—Quadriceps; Tgfbr1KO/Acvr1bKO/Acvr: TgfbKO—mouse lines that Tgfbr1, Acvr1b or both receptors were deleted; h—hours; d—days; CKD/CKD + 4PBA—Chronic kidney disease mice group that were given a modified diet with or without 4-PBA; KPC/KPC IL—mice underwent orthotopic implantation of KPC or KPC IL6KO cells; IM + GYY/IM + NaHS—mice were immobilized and injected with GYY4137 or NaHS; IM(gyy)/IM(nahs)—mice were immobilized and utilized as the control group for IM + GYY or IM + NaHS.

## Appendix B

### Appendix B.1

**Table A1:** Raw data extracted from the 29 studies (Cross-Sectional Area).

Cohorts	Studies	n_ctrl	CSA_ctrl (μm²)	SD_ctrl (μm²)	n_injury	CSA_injury (μm²)	SD_injury (μm²)
1	Hillege et al. [13] (TA)_Acvr1bKO	6.0	423.82	94.991	6.0	330.47	122.132
2	Hillege et al. [13] (TA)_Tgfbr1KO	6.0	423.82	94.991	6.0	294.18	230.693
3	Hillege et al. [13] (TA)_Acvr:TgfbKO	6.0	423.82	94.991	6.0	532.96	244.263
4	Niida et al. [14] (GA)_CKD	5.0	2014.16	125.399	5.0	1134.38	50.043
5	Niida et al. [14] (GA)_CKD + 4PBA	5.0	2014.16	125.399	5.0	1543.86	125.399
6	Xu et al. [15] (TA)_IM(gyy)	6.0	1469.09	260.454	6.0	823.03	173.007
7	Xu et al. [15] (TA)_IM + GYY	6.0	1469.09	260.454	6.0	1221.11	217.196
8	Xu et al. [15] (TA)_IM(nahs)	8.0	1442.61	300.747	8.0	814.2	224.747
9	Xu et al. [15] (TA)_IM + NaHS	8.0	1442.61	300.747	8.0	1185.79	400.647
10	Li et al. [16] (TA)_TNF	8.0	2488.72	938.43	8.0	980.25	293.23
11	Li et al. [16] (TA)_TRAF	8.0	2488.72	938.43	6.0	2271.16	636.74
12	Li et al. [16] (TA)_TNF:TRAF	8.0	2488.72	938.43	6.0	1734.38	259.87
13	Zheng et al. [17] (TA)_Day1	5.0	1024.56	171.797	5.0	916.56	92.931
14	Zheng et al. [17] (TA)_Day3	5.0	1111.77	187.271	5.0	613.35	156.279
15	Zheng et al. [17] (TA)_Day5	5.0	1108.31	140.805	5.0	596.03	54.918
16	Lee et al. [18] (QUA)_TC	4.0	1664.65	87.480	4.0	1365.76	84.940
17	Lee et al. [18] (QUA)_A5	4.0	1664.65	87.480	4.0	1579.39	129.320
18	Lee et al. [18] (QUA)_A10	4.0	1664.65	87.480	4.0	1877.97	87.480
19	Keeble et al. [19] (QUA)_6 h	6.0	2355.43	548.51	6.0	2463.02	451.48
20	Keeble et al. [19] (QUA)_1 d	6.0	2011.53	301.63	6.0	2129.68	806.92
21	Keeble et al. [19] (QUA)_3 d	6.0	2398.66	743.52	6.0	2150.82	720.46
22	Keeble et al. [19] (QUA)_7 d	6.0	2194.04	171.95	6.0	2000	274.74
23	Ozaki et al. [20] (GA)_DEX	4.0	1098.46	41.020	4.0	850.59	205.120
24	Rupert et al. [21] (QUA)_KPC	4.0	952.13	258.68	4.0	645.58	236.92
25	Rupert et al. [21] (QUA)_KPC IL	4.0	952.13	258.68	4.0	997.72	380.62
26	Sun et al. [22] (TA)	9.0	657.49	55.72	9.0	479.12	40.25
27	Polge et al. [23] (TA)_DEX 9	4.5	5.66	3.967	4.5	4.14	2.227
28	Polge et al. [23] (TA)_DEX 14	4.5	5.66	3.967	4.5	4.39	3.585
29	Hwee et al. [24] (GA)	5.0	1203.71	737.679	5.0	1680.9	266.852
30	Zheng et al. [25] (GA)	12.0	2247.05	254.230	12.0	2042.35	162.986
31	Chen et al. [26] (GA)	3.0	3228.5	2359.24	3.0	1339.22	952.84
32	Saul et al. [27] (QUA)	8.0	3970.4	236.174	8.0	901.69	553.127
33	Moarbes et al. [28] (TA)_24 h	8.0	613.73	138.508	8.0	274.95	76.594
34	Moarbes et al. [28] (TA)_48 h	8.0	552.13	124.875	8.0	206.3	69.240
35	Moarbes et al. [28] (TA)_96 h	8.0	611.13	132.229	8.0	191.46	76.594
36	Chen et al. [29] (GA)	6.0	589.68	143.64	6.0	268.69	81.27
37	Hirata et al. [30] (TA)	4.0	2032.4	150.800	4.0	1243.3	101.200
38	Giordano et al. [31] (DIA)	5.0	1313.5	564.607	5.0	933.5	131.034
39	de Theije et al. [32] (GA)	8.0	2177.19	374.795	8.0	1852.45	204.778
40	Kawanishi et al. [33] (GA)	5.5	1797.87	365.899	5.5	1079.19	202.345
41	Ono et al. [34] (GA)	10.0	2059.04	355.883	10.0	1226.93	75.831
42	Jaitovich et al. [35] (SOL)	4.5	14,618.62	174,981.861	4.5	8596.9	491,930.137
43	Files et al. [36] (EDL)	5.0	1621.02	77.793	5.0	1236	257.081
44	Zhang et al. [37] (GA)	8.0	64,680.6	10,758.6	8.0	32,946.6	5624.4
45	Cunha et al. [38] (PLA)	6.0	2039.34	505.942	6.0	1431.14	265.010
46	Haegens et al. [39] (DIA)	6.0	58,684.9	6622.35	6.0	48,480.6	6330.8
47	Onda et al. [40] (SOL)	7.0	1840.84	317.252	7.0	1029.06	115.355
48	Ibebunjo et al. [41] (TRI)	6.0	92,392.5	16,487.515	6.0	80,200.5	10,265.812

PLA—plantaris; EDL—extensor digitorum longus; DIA—diaphragm; TA—tibialis anterior; TRI—triceps; SOL—soleus; QUA—quadriceps; Tgfbr1KO/Acvr1bKO/Acvr:TgfbKO—mouse lines in which Tgfbr1, Acvr1b or both receptors were deleted; h—hours; d—days; CKD/CKD + 4PBA—chronic kidney disease mice group that were given a modified diet with or without 4-PBA; KPC/KPC IL—mice that underwent orthotopic implantation of KPC or KPC IL6KO cells; IM + GYY/IM + NaHS—mice that were immobilized and injected with GYY4137 or NaHS; IM(gyy)/IM(nahs)—mice that were immobilized and utilized as the control group for IM + GYY or IM + NaHS; n_ctrl—sample size of the control condition; n_injury—sample size of the injury condition; CSA_ctrl—average cross-sectional area of the control condition; CSA_injury—average cross-sectional area of the injury condition; SD_ctrl—standard deviation of the control condition; SD_injury—standard deviation of the injury condition.

### Appendix B.2

**Table A2:** Raw data extracted from the 29 studies (MAFbx mRNA).

Cohorts	Studies	n_ctrl	MAFbx_ctrl (Relative Levels)	SD_ctrl (Relative Levels)	n_injury	MAFbx_injury (Relative Levels)	SD_injury (Relative Levels)
1	Hillege et al. [13] (TA)_Acvr1bKO	6.0	0.088	0.152	6.0	0.088	0.135
2	Hillege et al. [13] (TA)_Tgfbr1KO	6.0	0.088	0.152	6.0	0.088	0.066
3	Hillege et al. [13] (TA)_Acvr:TgfbKO	6.0	0.088	0.152	6.0	0.29	0.255
4	Niida et al. [14] (GA)_CKD	5.0	0.98	1.14	5.0	2.58	2.594
5	Niida et al. [14] (GA)_CKD + 4PBA	5.0	0.98	1.14	5.0	1.35	1.006
6	Xu et al. [15] (TA)_IM(gyy)	6.0	0.803	1.2	6.0	5.05	4.777
7	Xu et al. [15] (TA)_IM + GYY	6.0	0.803	1.2	6.0	2.29	3.677
8	Xu et al. [15] (TA)_IM(nahs)	8.0	0.9	2.404	8.0	5.202	5.091
9	Xu et al. [15] (TA)_IM + NaHS	8.0	0.9	2.404	8.0	1.95	4.384
10	Li et al. [16] (TA)_TNF	8.0	1.05	0.86	8.0	2.17	1.06
11	Li et al. [16] (TA)_TRAF	8.0	1.05	0.86	6.0	0.89	0.29
12	Li et al. [16] (TA)_TNF:TRAF	8.0	1.05	0.86	6.0	0.72	0.68
13	Zheng et al. [17] (TA)_Day1	5.0	0.95	0.52	5.0	3.84	0.96
14	Zheng et al. [17] (TA)_Day3	5.0	0.98	0.58	5.0	1.52	1.34
15	Zheng et al. [17] (TA)_Day5	5.0	1.02	0.64	5.0	1.52	1.68
16	Lee et al. [18] (QUA)_TC	4.0	0.96	1.006	4.0	4.85	5.121
17	Lee et al. [18] (QUA)_A5	4.0	0.96	1.006	4.0	1.73	1.766
18	Lee et al. [18] (QUA)_A10	4.0	0.96	1.006	4.0	1.29	3.198
19	Keeble et al. [19] (QUA)_6 h	6.0	1	0.79	6.0	0.93	0.61
20	Keeble et al. [19] (QUA)_1 d	6.0	1	0.78	6.0	1.62	0.57
21	Keeble et al. [19] (QUA)_3 d	6.0	1.07	1.33	6.0	2.2	2.52
22	Keeble et al. [19] (QUA)_7 d	6.0	1.02	0.7	6.0	0.92	0.25
23	Ozaki et al. [20] (GA)_DEX	4.0	1.01	0.514	4.0	2.96	3.87
24	Rupert et al. [21] (QUA)_KPC	4.0	1.13	1.7	4.0	17.65	3.46
25	Rupert et al. [21] (QUA)_KPC IL	4.0	1.13	1.7	4.0	3.65	4.4
26	Sun et al. [22] (TA)	9.0	1	0.081	9.0	3.1	0.768
27	Polge et al. [23] (TA)_DEX 9	4.5	1.7	2.715	4.5	4.63	6.64
28	Polge et al. [23] (TA)_DEX 14	4.5	1.7	2.715	4.5	4.35	5.091
29	Hwee et al. [24] (GA)	5.0	1.34	2.12	5.0	2.34	2.3
30	Zheng et al. [25] (GA)	12.0	0.99	0.686	12.0	1.46	0.682
31	Chen et al. [26] (GA)	3.0	1.02	0.92	3.0	3.95	4.05
32	Saul et al. [27] (QUA)	8.0	2.26	0.933	8.0	15.41	11.399
33	Moarbes et al. [28] (TA)_24 h	8.0	0.86	0	8.0	13.63	5.996
34	Moarbes et al. [28] (TA)_48 h	8.0	0.86	0	8.0	6.28	3.564
35	Moarbes et al. [28] (TA)_96 h	8.0	0.86	0	8.0	21.5	5.798
36	Chen et al. [29] (GA)	6.0	1.52	0.37	6.0	20.64	2.51
37	Hirata et al. [30] (TA)	4.0	19.05	4.24	4.0	24.01	1.72
38	Giordano et al. [31] (DIA)	5.0	1	0.16	5.0	1.17	0.16
39	de Theije et al. [32] (GA)	8.0	6.42	2.432	8.0	21.3	4.13
40	Kawanishi et al. [33] (GA)	5.5	2.05	0.61	5.5	12.96	8.841
41	Ono et al. [34] (GA)	10.0	4.23	2.308	10.0	22.3	1.897
42	Jaitovich et al. [35] (SOL)	4.5	-	-	4.5	-	-
43	Files et al. [36] (EDL)	5.0	0.79	0.447	5.0	12.9	9.168
44	Zhang et al. [37] (GA)	8.0	2.45	0.33	8.0	22.75	4.1
45	Cunha et al. [38] (PLA)	6.0	8.76	0	6.0	14.02	1.788
46	Haegens et al. [39] (DIA)	6.0	4.63	0.53	6.0	12.24	4.17
47	Onda et al. [40] (SOL)	7.0	4.3	1.746	7.0	13.03	1.931
48	Ibebunjo et al. [41] (TRI)	6.0	3.77	0.49	6.0	3.64	0.49

PLA—plantaris; EDL—extensor digitorum longus; DIA—diaphragm; TA—tibialis anterior; TRI—triceps; SOL—soleus; QUA—quadriceps; Tgfbr1KO/Acvr1bKO/Acvr:TgfbKO—mouse lines in which Tgfbr1, Acvr1b or both receptors were deleted; h—hours; d—days; CKD/CKD + 4PBA—chronic kidney disease mice group that were given a modified diet with or without 4-PBA; KPC/KPC IL—mice that underwent orthotopic implantation of KPC or KPC IL6KO cells; IM + GYY/IM + NaHS—mice that were immobilized and injected with GYY4137 or NaHS; IM(gyy)/IM(nahs)—mice that were immobilized and utilized as the control group for IM + GYY or IM + NaHS; n_ctrl—sample size of the control condition; n_injury—sample size of the injury condition; MAFbx_ctrl—relative levels of the control condition; MAFbx_injury—relative levels area of the injury condition; SD_ctrl—standard deviation of the control condition; SD_injury—standard deviation of the injury condition.

### Appendix B.3

**Table A3:** Raw data extracted from the 29 studies (MuRF-1 mRNA).

Cohorts	Studies	n_ctrl	MuRF-1_ctrl (Relative Levels)	SD_ctrl (Relative Levels)	n_injury	MuRF-1_injury (Relative Levels)	SD_injury (Relative Levels)
1	Hillege et al. [13] (TA)_Acvr1bKO	6.0	0.19	0.118	6.0	0.19	0.149
2	Hillege et al. [13] (TA)_Tgfbr1KO	6.0	0.19	0.118	6.0	0.19	0.083
3	Hillege et al. [13] (TA)_Acvr:TgfbKO	6.0	0.19	0.118	6.0	0.28	0.203
4	Niida et al. [14] (GA)_CKD	5.0	1	1.297	5.0	3.27	3.935
5	Niida et al. [14] (GA)_CKD + 4PBA	5.0	1	1.297	5.0	1.67	1.409
6	Xu et al. [15] (TA)_IM(gyy)	6.0	1.099	1.347	6.0	5.102	5.511
7	Xu et al. [15] (TA)_IM + GYY	6.0	1.099	1.347	6.0	2.25	3.16
8	Xu et al. [15] (TA)_IM(nahs)	8.0	1.099	2.271	8.0	4.6	7.354
9	Xu et al. [15] (TA)_IM + NaHS	8.0	1.099	2.271	8.0	2.4	5.063
10	Li et al. [16] (TA)_TNF	8.0	0.98	0.22	8.0	4.13	1.86
11	Li et al. [16] (TA)_TRAF	8.0	0.98	0.22	8.0	2.63	1.27
12	Li et al. [16] (TA)_TNF:TRAF	8.0	0.98	0.22	8.0	1.56	2.16
13	Zheng et al. [17] (TA)_Day1	5.0	0.96	0.64	5.0	3.38	2.32
14	Zheng et al. [17] (TA)_Day3	5.0	0.96	0.44	5.0	0.87	0.7
15	Zheng et al. [17] (TA)_Day5	5.0	0.96	0.28	5.0	0.87	0.19
16	Lee et al. [18] (QUA)_TC	4.0	1	1.342	4.0	7.53	12.947
17	Lee et al. [18] (QUA)_A5	4.0	1	1.342	4.0	2.49	4.025
18	Lee et al. [18] (QUA)_A10	4.0	1	1.342	4.0	1.69	2.214
19	Keeble et al. [19] (QUA)_6 h	6.0	0.96	0.32	6.0	0.82	0.54
20	Keeble et al. [19] (QUA)_1 d	6.0	1.05	0.98	6.0	1.66	0.52
21	Keeble et al. [19] (QUA)_3 d	6.0	1	0.74	6.0	3.33	2.75
22	Keeble et al. [19] (QUA)_7 d	6.0	1.07	1.61	6.0	1.26	2.39
23	Ozaki et al. [20] (GA)_DEX	4.0	1.06	0.563	4.0	2.92	3.87
24	Rupert et al. [21] (QUA)_KPC	4.0	1.13	1.6	4.0	19.95	7.77
25	Rupert et al. [21] (QUA)_KPC IL	4.0	1.13	1.6	4.0	1.44	1.14
26	Sun et al. [22] 14 (TA)	9.0	1	0.09	9.0	3.72	0.67
27	Polge et al. [23] (TA)_DEX 9	4.5	1.73	3.649	4.5	4.23	4.582
28	Polge et al. [23] (TA)_DEX 14	4.5	1.73	3.649	4.5	3.14	3.967
29	Hwee et al. [24] (GA)	5.0	1.37	2.08	5.0	0.05	0.1
30	Zheng et al. [25] (GA)	12.0	1.003	0.26	12.0	1.627	0.197
31	Chen et al. [26] (GA)	3.0	0.96	0.846	3.0	3.45	3.4
32	Saul et al. [27] (QUA)	8.0	3.11	1.131	8.0	16.14	13.096
33	Moarbes et al [28] (TA)_24 h	8.0	0.86	0	8.0	20.64	7.863
34	Moarbes et al [28] (TA)_48 h	8.0	0.86	0	8.0	7.14	2.63
35	Moarbes et al [28] (TA)_96 h	8.0	0.86	0	8.0	12.5	4.299
36	Chen et al. [29] (GA)	6.0	1.82	0.4	6.0	17.56	3.8
37	Hirata et al. [30] (TA)	4.0	18.79	0.8	4.0	24.41	1.98
38	Giordano et al. [31] (DIA)	5.0	1	0.54	5.0	1.05	29.695
39	de Theije et al. [32] (GA)	8.0	8.6	1.499	8.0	19.38	310.005
40	Kawanishi et al. [33] (GA)	5.5	2.05	0.61	5.5	9.26	4.644
41	Ono et al. [34] (GA)	10.0	7.21	3.131	10.0	24.01	3.763
42	Jaitovich et al. [35] (SOL)	4.5	3.44	0	4.5	15.74	7.573
43	Files et al. [36] (EDL)	5.0	1.06	5.188	5.0	13.23	10.196
44	Zhang et al. [37] (GA)	8.0	3.24	0.33	8.0	22.75	4.1
45	Cunha et al. [38] (PLA)	6.0	8.76	0	6.0	11.64	3.552
46	Haegens et al. [39] (DIA)	6.0	2.32	1.12	6.0	12.44	8.2
47	Onda et al. [40] (SOL)	7.0	5.49	2.275	7.0	13.36	5.424
48	Ibebunjo et al. [41] (TRI)	6.0	5.56	0.637	6.0	12.54	1.127

PLA—plantaris; EDL—extensor digitorum longus; DIA—diaphragm; TA—tibialis anterior; TRI—triceps; SOL—soleus; QUA—quadriceps; Tgfbr1KO/Acvr1bKO/Acvr:TgfbKO—mouse lines in which Tgfbr1, Acvr1b or both receptors were deleted; h—hours; d—days; CKD/CKD + 4PBA—chronic kidney disease mice group that were given a modified diet with or without 4-PBA; KPC/KPC IL—mice that underwent orthotopic implantation of KPC or KPC IL6KO cells; IM + GYY/IM + NaHS—mice that were immobilized and injected with GYY4137 or NaHS; IM(gyy)/IM(nahs)—mice that were immobilized and utilized as the control group for IM + GYY or IM + NaHS; n_ctrl—sample size of the control condition; n_injury—sample size of the injury condition; MuRF-1_ctrl—relative levels of the control condition; MuRF-1_injury—relative levels of the injury condition; SD_ctrl—standard deviation of the control condition; SD_injury—standard deviation of the injury condition.
